# Lifecycles of Cochrane Systematic Reviews (2003–2024): A Bibliographic Study

**DOI:** 10.1002/cesm.70043

**Published:** 2025-08-17

**Authors:** Shiyin Li, Chong Wu, Zichen Zhang, Mengli Xiao, Mohammad Hassan Murad, Lifeng Lin

**Affiliations:** ^1^ Department of Epidemiology and Biostatistics University of Arizona Tucson AZ USA; ^2^ Department of Biostatistics The University of Texas MD Anderson Cancer Center Houston TX USA; ^3^ Institute for Data Science in Oncology The University of Texas MD Anderson Cancer Center Houston TX USA; ^4^ Department of Biostatistics and Informatics University of Colorado Anschutz Medical Campus Aurora CO USA; ^5^ Evidence‐Based Practice Center Mayo Clinic Rochester MN USA

**Keywords:** Cochrane systematic reviews, lifecycle, meta‐research, review update, withdrawal

## Abstract

**Background and Objectives:**

The relevance of Cochrane systematic reviews depends on timely completion and updates. This study aimed to empirically assess the lifecycles of Cochrane reviews published from 2003 to 2024, including transitions from protocol to review, update patterns, and withdrawals.

**Methods:**

We extracted data from Cochrane Library publications between 2003 and 2024. Each review topic was identified using a unique six‐digit DOI‐based ID. We recorded protocol publication, review publication, updates, and withdrawals (i.e., removed from the Cochrane Library for editorial or procedural reasons), calculating time intervals between stages and conducting subgroup analyses by review type.

**Results:**

Of 8137 protocols, 71.9% progressed to reviews (median 25.7 months), 2.4% were updated during the protocol stage, and 10.0% were withdrawn. Among 8477 reviews, 64.3% were never updated by the time of our analysis; for those updated at least once, the median interval between updates was 57.2 months. Withdrawal occurred in 2.5% of reviews (median 67.6 months post‐publication). Subgroup analyses showed variation across review types; diagnostic and qualitative reviews tended to have longer protocol‐to‐review times than other types of reviews.

**Conclusions:**

Cochrane reviews show long development and update intervals, with variation by review type. Greater use of automation and targeted support may improve review efficiency and timeliness.

## 1 Introduction

Systematic reviews are essential tools in healthcare, synthesizing evidence to inform clinical practice, guideline development, and policy decisions. Cochrane systematic reviews, hosted in the Cochrane Library, are recognized for their rigorous methodology and structured updating processes and are often considered the “gold standard.” [1, 2, 3] Cochrane systematic reviews typically start as detailed protocols that progress to published reviews, subsequently updated to maintain relevance and accuracy over time. Timely updates are critical, yet maintaining a balance between methodological rigor and speed remains challenging, especially given the increasing availability of automated and AI‐assisted review methodologies [4, 5, 6].

Previous studies have demonstrated variability in the time taken from protocol to full review, update frequency, and withdrawal rates within Cochrane systematic reviews [7, 8]. However, few comprehensive empirical analyses have tracked entire lifecycles, including protocols, published reviews, updates, and withdrawals, over extended periods. This study aims to comprehensively evaluate the lifecycles of a large collection of Cochrane systematic reviews, analyzing key intervals from protocols to reviews, update patterns, and subgroup differences across review types. By providing a detailed characterization, we aim to inform future strategies for the production and maintenance of systematic reviews, particularly the integration of automated technologies.

## 2 Methods

We systematically extracted data from publicly available records in the Cochrane Library, spanning Issue 1 of 2003 to Issue 12 of 2024. We selected 2003 as the starting point because the Cochrane Library began systematically indexing reviews and protocols with complete metadata from that year onward. This period also represents the majority of Cochrane systematic reviews, ensuring a comprehensive and representative data set. We identified each publication via its unique Digital Object Identifier (DOI). Publications were categorized as protocols, published reviews, updated protocols or reviews, or withdrawal notices. Editorials and methodological articles were excluded.

We used R (version 4.5.1) with the “rvest” package (version 1.0.4) to extract DOI information and publication dates from Cochrane Library webpages. We then cleaned and reshaped the data using the “dplyr” package (version 1.1.4) and performed date calculations with the “lubridate” package (version 1.9.4). Data organization and management were conducted using Microsoft Excel. Each Cochrane systematic review topic has a unique identifier in the format CDXXXXXX (a “CD” prefix followed by six numerical digits), embedded in its DOI, enabling comprehensive lifecycle tracking. For each ID, we documented whether the topic originated as a protocol, whether it transitioned into a published review, how many updates it underwent and when, and whether it was eventually withdrawn and at what point in the timeline.

We calculated intervals (in months) between critical stages, including from the last protocol publication to the first published review, intervals between review updates, and from the last review publication to withdrawal (if applicable). We calculated descriptive statistics, including medians, means, interquartile ranges (IQRs), and percentages. We also conducted subgroup analyses based on review type: diagnostic, intervention, overview, qualitative, rapid, prognosis, and prototype.

## 3 Results

**Figure 1:**
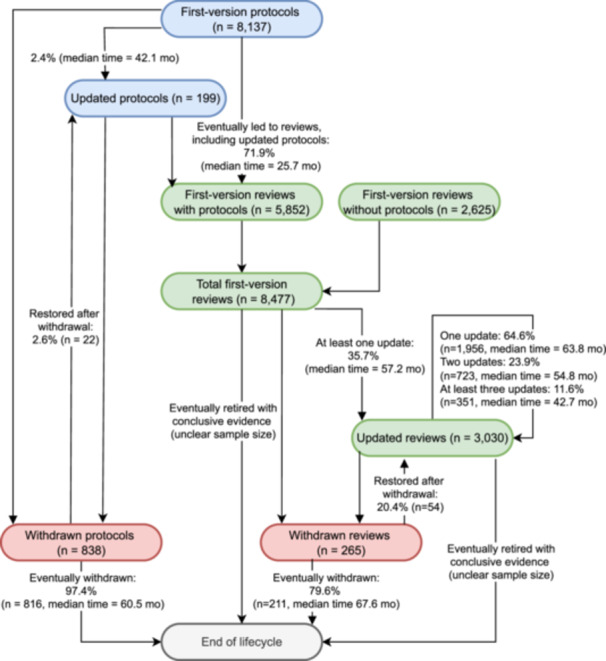
Diagram illustrating the lifecycles of Cochrane systematic reviews. *Note:* Cochrane systematic reviews do not routinely specify whether further updates will be undertaken or if the evidence is considered conclusive. As a result, information on reviews retired due to conclusiveness is largely unavailable, although most reviews will eventually cease to be updated.

Figure 1 illustrates the lifecycles of Cochrane systematic reviews, with detailed results presented in Table 1. The Cochrane Library included 8137 records of protocols, of which 2.4% were updated during the protocol stage, while 10.0% were eventually withdrawn (median time = 60.5 months from the most recent protocol to withdrawal). Of note, this count reflects all protocol versions published between 2003 and 2024, including multiple versions for the same topic, such as protocols that were subsequently updated, withdrawn, or progressed to completed reviews. Additionally, 71.9% of protocols progressed to a full review, with a median transition time of 25.7 months (IQR 15.3–42.0), consistent with prior research reporting a median of approximately 2.4 years [7].

**Table 1:** Descriptive statistics of Cochrane systematic reviews, protocols, and update time gaps.

Type (Count)	Stage	Count (Percentage)	Median Time (Month)	IQR^a^ (Month)	Mean Time (Month)
Protocols (8137)	With no updates	7938 (97.6%)	NA	NA	NA
	With at least 1 update^b^	199 (2.4%)	42.1	18.5–68.2	47.4 ± 36.5
	Eventually led to reviews^c^	5852 (71.9%)	25.7	15.3–42.0	32.8 ± 25.5
	Eventually withdrawn^d^	816 (10.0%)	60.5	37.9–95.5	69.4 ± 41.5
Reviews (8477)	Overall^e^	NA	57.2	37.5–82.3	64.1 ± 36.8
	With no update	5447 (64.3%)	NA	NA	NA
	With 1 update^b^	1956 (23.1%)	63.8	38.8–94.6	70.4 ± 41.7
	With 2 updates^b^	723 (8.5%)	54.8	39.2–72.0	56.7 ± 22.7
	With at least 3 updates^b^	351 (4.1%)	42.7	32.7–54.1	44.1 ± 15.4
	Eventually withdrawn^d^	211 (2.5%)	67.6	47.8–102.9	73.1 ± 40.5

Abbreviation: NA, not applicable.

^a^ Interquartile range.

^b^ For protocols or reviews with exactly one update, the update time is calculated as the difference between the date of the last version and the date of the first version. For protocols or reviews with two or more updates, the (averaged) update time is calculated as the difference between the date of the last version and the date of the first version, divided by the number of updates.

^c^ Time from the publication of the final protocol version to the publication of the first full review.

^d^ Time from the publication of the final protocol or review version to the withdrawal date, excluding those reviews that had an additional protocol or review published after withdrawal.

^e^ Calculated among reviews that underwent at least one update.

Table 2 presents the results of the most frequent subgroups of Cochrane reviews. The median transition times were 31.1 months for diagnostic reviews, 25.6 for intervention reviews, 22.0 for overviews, 32.6 for qualitative reviews, 24.0 for prognosis reviews, and 22.1 for prototype reviews.

**Table 2:** Descriptive statistics for subgroups of intervention, diagnostic, overview, prognosis, prototype, qualitative, and rapid reviews.

Subgroup	Type (Count)	Stage	Count (Percentage)	Median Time (Month)	IQR (Month)	Mean time ± SD (Month)
Intervention	Protocols (7705)	With no updates	7511 (97.5%)	NA	NA	NA
		With at least 1 update	194 (2.5%)	42.6	18.3–69.1	47.6 ± 36.8
		Eventually led to reviews	5596 (72.6%)	25.6	15.2–42.0	32.7 ± 25.7
		Eventually withdrawn	782 (10.1%)	61.0	37.8–95.2	69.6 ± 41.6
	Reviews (8141)	With no update	5154 (63.3%)	NA	NA	NA
		With at least 1 update	2987 (36.7%)	57.3	50.0–123.2	64.3 ± 36.8
		Eventually withdrawn	210 (2.6%)	67.7	47.8–103.0	73.2 ± 40.5
Diagnostic	Protocols (248)	With no updates	245 (98.8%)	NA	NA	NA
		With at least 1 update	3 (1.1%)	43.2	30.7–61.2	46.9 ± 30.7
		Eventually led to reviews	154 (62.1%)	31.1	18.8–48.0	36.8 ± 23.9
		Eventually withdrawn	25 (10.1%)	54.1	47.1–102.4	70.4 ± 38.4
	Reviews (192)	With no update	163 (84.9%)	NA	NA	NA
		With at least 1 update	29 (15.1%)	55.8	28.7–77.3	54.2 ± 31.1
		Eventually withdrawn	0 (0.0%)	—	—	—
Overview	Protocols (87)	With no updates	85 (97.7%)	NA	NA	NA
		With at least 1 update	2 (2.3%)	32.5	—	32.5 ± 2.1
		Eventually led to reviews	63 (72.4%)	22.0	11.3–37.0	26.7 ± 21.3
		Eventually withdrawn	7 (8.0%)	51.0	33.5–78.5	56.4 ± 32.3
	Reviews (70)	With no update	63 (90.0%)	NA	NA	NA
		With at least 1 update	7 (10.0%)	35.6	28.7–77.3	42.2 ± 41.9
		Eventually withdrawn	1 (1.4%)	62.6	—	—
Prognosis	Protocols (47)	With no updates	47 (100.0%)	NA	NA	NA
		With at least 1 update	0 (0.0%)	—	—	—
		Eventually led to reviews	20 (42.6%)	24.0	14.1–38.4	27.8 ± 17.0
		Eventually withdrawn	0 (0.0%)	—		
	Reviews (21)	With no update	20 (95.2%)	NA	NA	NA
		With at least 1 update	1 (4.8%)	3.9	—	—
		Eventually withdrawn	0 (0.0%)	—	—	—
Prototype	Protocols (8)	With no updates	8 (100.0%)	NA	NA	NA
		With at least 1 update	0 (0.0%)	—	—	—
		Eventually led to reviews	3 (37.5%)	22.1	—	18.3 ± 8.1
		Eventually withdrawn	0 (0.0%)	—	—	—
	Reviews (11)	With no update	10 (90.9%)	NA	NA	NA
		With at least 1 update	1 (9.1%)	30.2	—	—
		Eventually withdrawn	0 (0.0%)	—	—	—
Qualitative	Protocols (41)	With no updates	41 (100.0%)	NA	NA	NA
		With at least 1 update	0 (0.0%)	—	—	—
		Eventually led to reviews	25 (61.0%)	32.6	19.7–38.9	33.0 ± 16.8
		Eventually withdrawn	1 (2.4%)	3.3	—	—
	Reviews (30)	With no update	29 (96.7%)	NA	NA	NA
		With at least 1 update	1 (3.3%)	0.3	—	—
		Eventually withdrawn	0 (0.0%)	—	—	—
Rapid	Protocols (1)	With no updates	1 (100.0%)	NA	NA	NA
		With at least 1 update	0 (0.0%)	—	—	—
		Eventually led to reviews	1 (100.0%)	4.3	—	—
		Eventually withdrawn	0 (0.0%)	—	—	—
	Reviews (13)	With no update	10 (76.9%)	NA	NA	NA
		With at least 1 update	3 (23.1%)	17.0	—	16.9 ± 10.6
		Eventually withdrawn	0 (0.0%)	—	—	—

*Note:* Some results are not reported due to the absence or limited availability of data. See the notes in Table 1 for details.

Beyond the protocol stage, the Cochrane Library documented 8477 reviews, of which 64.3% were never updated by the time of our analysis, 23.1% had one update, 8.5% had two updates, and 4.1% had three or more updates. Among reviews that underwent at least one update, the median time between updates was 57.2 months. We report these results stratified by the number of updates (i.e., 1 update, 2 updates, or at least 3 updates) in Table 1, which reflects the natural decrease in sample size with each successive update.

In addition, 211 reviews (2.5%) were ultimately withdrawn, with a median time of 67.6 months from the final review publication to withdrawal. In 54 cases, the withdrawal notice preceded the nominal “final” review date, likely due to editorial arrangements.

## 4 Discussion

### 4.1 Implications

This large‐scale empirical analysis demonstrates substantial delays and variability within Cochrane systematic review lifecycles, particularly in protocol‐to‐review transitions and subsequent updates. While longer intervals do not inherently mean that systematic reviews are out‐of‐date, since some topics may not warrant frequent updates, they do highlight the potential risk of lagging behind new evidence, especially in rapidly evolving fields. Our findings align with prior research indicating that many systematic reviews may not keep pace with the rate of new evidence generation [7, 8], raising concerns about their continued practical relevance and applicability in some contexts.

The long median intervals identified raise concerns about the potential obsolescence of reviews, especially in rapidly evolving clinical fields. Although Cochrane policies strongly encourage regular updating, our findings suggest that policy alone is insufficient to ensure timely reviews [9]. Automation and integration of AI technologies could substantially reduce manual workload, streamline updates, and improve the responsiveness of reviews to emerging evidence [10, 11]. Recent advances in AI demonstrate its potential to expedite critical review processes, such as literature screening and data extraction, significantly reducing update intervals [12, 13]. However, while AI shows promise in facilitating the conduct of new systematic reviews, its role in accelerating updates remains less certain due to the complexities of integrating new evidence into existing syntheses and reassessing conclusions [14]. Future research should explore how AI tools might be adapted or developed to address these specific challenges in the updating process.

Our subgroup analyses identified variations in review lifecycles based on review type. Diagnostic and qualitative reviews had notably longer transitions from protocol to review publication. This may not necessarily reflect more complex methodologies, but could instead be due to the greater need for manual effort and human judgment, such as assessing nuanced diagnostic criteria or synthesizing nonquantitative data. Recognizing these labor‐intensive demands, Cochrane and systematic review groups could implement tailored strategies to complement existing resources and infrastructure. These might include expanding capacity, improving workflow efficiency, and developing novel tools such as semi‐automated data extraction or AI‐assisted qualitative synthesis to reduce manual effort and further improve lifecycle efficiency.

In parallel with technological innovations, Cochrane has also introduced focused review formats with simplified reporting requirements to help reduce the workload for authors and editors. These formats may complement automation efforts by allowing for more targeted, question‐specific reviews that can be completed and updated more efficiently, particularly when rapid evidence synthesis is needed.

### 4.2 Strengths and Limitations

A strength of this study is its extensive scope, covering over two decades and more than 22,000 Cochrane publications (including different versions), enabling robust characterization of lifecycle patterns. However, limitations include reliance on publicly available data, which lack detailed contextual information such as internal editorial decisions, communication with authors, resource constraints, or topic prioritization processes. Without access to these internal factors, it is difficult to determine why some reviews are delayed, never completed, not updated, or withdrawn. Further qualitative research could illuminate these internal dynamics, helping to design more effective strategies for review management.

In addition to qualitative approaches, further empirical research should consider analytical methods to assess the necessity and timing of updates relative to evolving evidence. Some reviews may remain valid without frequent updates, while others quickly become outdated [15, 16]. A lack of updates does not necessarily indicate inefficiency; in many cases, no new studies or no impactful evidence may have emerged to warrant revision. Differentiating between reviews that are outdated and those that remain current due to a stable evidence base is essential for prioritizing updates effectively. Cochrane has recently introduced a decision framework to guide authors and editors in determining when to update reviews, incorporating considerations such as evolving evidence, changes in interventions, and methodological advancements. Continued development of objective, data‐driven criteria could further support prioritization efforts, helping allocate limited resources more effectively across diverse review portfolios.

Future studies could also examine temporal trends, such as whether review development times and update frequencies have changed over successive 5‐year periods. This may reveal shifts in practices and efficiencies over time. Additionally, examining the outcomes and consequences of withdrawn reviews, including whether they are ever reinstated or revised, could provide deeper insights into systematic review lifecycle management and inform future editorial and methodological policies.

## 5 Conclusions

Our study highlights the need for improved methods to manage the lifecycle of Cochrane systematic reviews. Automated methods and AI could notably enhance efficiency, reduce delays, and ensure timely integration of emerging evidence, thereby maintaining the relevance and clinical utility of systematic reviews. Addressing methodological complexity through tailored strategies, clarifying update criteria, and exploring internal review dynamics could further optimize future systematic review production.

## Author Contributions

**Shiyin Li:** methodology, formal analysis, investigation, writing – original draft. **Chong Wu:** data curation, writing – review and editing, funding acquisition. **Zichen Zhang:** data curation, writing – review and editing. **Mengli Xiao:** writing – review and editing. **Mohammad Hassan Murad:** writing – review and editing. **Lifeng Lin:** conceptualization, methodology, data curation, writing – review and editing, supervision, funding acquisition.

## Ethics Statement

This study is based on publicly available Cochrane systematic reviews and does not involve human participants or identifiable personal data. Therefore, it was exempt from ethics committee approval.

## Conflicts of Interest

The authors declare no conflicts of interest.

## Peer Review

The peer review history for this article is available at https://www.webofscience.com/api/gateway/wos/peer-review/10.1002/cesm.70043.

## Data Availability

The data used in this manuscript are directly sourced from published Cochrane systematic reviews. They are also available from the corresponding author upon reasonable request.
